# Book Reviews

**Published:** 1899-05

**Authors:** 


### *BOOK REVIEWS, PAMPHLETS, EXCHANGES.*

### *BOOK REVIEWS.*

[Contributions solicited for review.]

A TREATISE ON FRACTURES AND DISLOCATIONS. For practitioners and students. By Lewis A. Stimson, B.A., M.D., Professor of Surgery in Connell University Medical College, New York. In one octavo volume of 823 pages, with 321 engravings and 20 full-page plates. Cloth, $5.00 *net.* Leather, $6.00 *net.* Just ready. Lee Brothers & Co., Philadelphia and New York.

This is the most classical work of its kind in the English language and will always be a standard text for reference for many years to come. To a surgeon whose work is at all of the emergency character, where fractures and dislocations are common, this work will prove of inestimable value. Dr. Stimson is peculiarly fitted for the writing of just such a work as this, for two reasons, viz.: Because, first, he is a man of considerable original aptitude, and secondly, because his opportunity for treating fractures and dislocations has been exceptionally large, in that for eleven years he has been the visiting surgeon to the Hudson Street Hospital, New York, a hospital where more such emergency cases are brought than to any other hospital probably in this country. The book is profusely illustrated, which adds much to its value, and the production of the skiagraphs, which occur throughout the book, makes this a most thoroughly up-to-date work. It is impossible to see how any surgeon would be without this book in his library.

---

NERVOUS AND MENTAL DISEASES. By Archibald Church, M.D., Professor of Clinical Neurology and Mental Diseases and Medical Jurisprudence in the Chicago Medical College, Chicago, and Frederick Peterson, M.D., Clinical Professor of Mental Diseases in the Woman's Medical College of New York. With 305 Illustrations. Published by W. B. Saunders, Philadelphia. Price $5.00 Cloth ; $6.00 half Morocco.

This work is an exceedingly clear exposition of neurology and psychiatry. There are several good works on nervous diseases by American authors, and this latest must certainly rank among the best. The preface states clearly just what this work proposes to do. "The literature of neurology and psychiatry has been sifted by the authors, and such digest revised in the light of their own experience in practice and in teaching. They have attempted to present their facts clearly, directly and with brevity despite the difficulty of condensing two great subjects within the limits of a single volume."

The preface further states, "this is not the work of two writers, but each author—Dr. Church in Neurology and Dr. Peterson in Psychiatry—has contributed to the making of a single volume what might have made a separate monograph; each is, therefore, solely responsible for the work in his own department."

The work throughout is written in an exceedingly clear style and the arrangements found eminently fit it for a text-book. The illustrations are numerous and excellent, and in the large majority of cases, are the originals of cases occurring in the author's practice. The work will no doubt find popular favor both among practitioners and students.

---

A TEXT-BOOK ON PRACTICAL OBSTETRICS. By Egbert H. Grandin, M.D., Gynecologist to the Columbus Hospital; Consulting Gynecologist to the French Hospital; late Consulting Obstetrician and Obstetric Surgeon of the New York Maternity Hospital; Fellow of the American Gynecological Society, etc. With the collaboration of George W. Jarman, M.D., Gynecologist to the Cancer Hospital; Instructor in Gynecology in the Medical Department of the Columbia University; late Obstetric Surgeon of the New York Maternity Hospital; Fellow of the American Gynecological Society, etc. Second Edition. Revised and Enlarged. Illustrated with Sixty-four Full-page Photographic Plates and Eighty-six Illustrations in the Text, 6½x9½ inches. Pages xiv–461. Extra Cloth, $4.00 net; Sheep, $4.75 net. The F. A. Davis Co., Publishers, 1914-16 Cherry St., Philadelphia.

This second edition has all to commend it which belonged to the first and it has besides several additions of marked value. Drs. Grandin and Jarman are certainly to be congratulated for this practical work which has been given to the profession. Every page contains something of real value, and there is no attempt to fill the book with verbosity. It is not what one would call an exhaustive treatise on obstetrics, but it is certainly a most practical one and contains just the things most needed. An exceedingly interesting feature of the book is the photographs taken from nature, *i. e.*, showing the actual delivery of the child and the after-birth, besides other features of obstetrical work. These illustrations add much to the work.

AN ESSAY ON THE NATURE AND THE CONSEQUENCES OF ANOMALIES OF REFRACTION. By F. C. Donders, M.D., Late Professor Ophthalmology in University of Utrecht. Revised and edited by Charles A. Oliver, A.M., M.D., of Philadelphia. Price $1.25. Published by P. Blakiston's Son & Co., Philadelphia.

Donders was a pioneer in ophthalmology. His name is inseparably connected with the subject of refraction, and its anomalies, and his writings show him to have been a close and accurate observer. His essay will be read with unusual interest. The fact that this book has been reviewed and edited by Charles A. Oliver, of Philadelphia, so well known as a writer, adds to its interest and value. The reviewer takes pleasure in recommending it to all students interested in refraction. The refined and cultivated taste of the publishers is very marked in its mechanical make-up. Its half Morocco bindings and green tints are very pleasing.

